# Antiviral and Antibacterial Effect of Honey Enriched with *Rubus* spp. as a Functional Food with Enhanced Antioxidant Properties

**DOI:** 10.3390/molecules27154859

**Published:** 2022-07-29

**Authors:** Dorota Grabek-Lejko, Michał Miłek, Ewelina Sidor, Czesław Puchalski, Małgorzata Dżugan

**Affiliations:** 1 Department of Bioenergetics, Food Analysis and Microbiology, Institute of Food Technology and Nutrition, University of Rzeszow, Zelwerowicza 4 St., 35-601 Rzeszow, Poland; cpuchalski@ur.edu.pl; 2 Department of Chemistry and Food Toxicology, Institute of Food Technology and Nutrition, University of Rzeszow, Ćwiklińskiej 1a St., 35-601 Rzeszow, Poland; mmilek@ur.edu.pl (M.M.); ewelina.sidor.dokt@gmail.com (E.S.); 3 Doctoral School, University of Rzeszow, Rejtana 16c, 35-959 Rzeszow, Poland

**Keywords:** honey, *Rubus*, blackberry, raspberry, fruit, leaves, antioxidants, antiviral, antibacterial

## Abstract

The aim of this study was to investigate the effect of blackberry and raspberry fruits (1 and 4%) and leaves (0.5 and 1%) on the biological activities of rape honey. Honey and plant material extracts were analyzed regarding total phenolic, flavonoid, anthocyanin contents, HPTLC and HPLC polyphenol profiles, as well as antioxidant activity. The antiviral potential was analyzed against bacteriophage phi 6—a coronavirus surrogate—whereas antimicrobial was tested against *S. aureus* and *E. coli*. Blackberry extracts were more abundant in antioxidants than raspberry extracts, with better properties found for leaves than fruits and for cultivated rather than commercial plants. The addition of both *Rubus* plant additives significantly increased the antioxidant potential of honey by four-fold (for 4% fruits additive) to five-fold (for 1% of leaves). Honey with the addition of fruits possessed higher antiviral potential compared with raw rape honey (the highest for 4% of raspberry fruit and 1% of blackberry leaf additive). Honey enriched with *Rubus* materials showed higher antibacterial potential against *S. aureus* than rape honey and effectively inhibited *S. aureus* biofilm formation. To summarize, honey enriched with *Rubus* fruit or leaves are characterized by increased pro-health value and can be recommended as a novel functional food.

## 1. Introduction

Consumers’ focus on health as well as knowledge about the influence of the food on our health has increased over the past few years. Food that can protect the body from different diseases is in great demand and the awareness of functional food is growing by around 8% annually [1]. Such food can be fortified with various additional ingredients, including antioxidants and phytochemicals, to increase a food’s health benefits. Ingredient interactions, known as synergism, can produce health benefits greater than the sum of the individual parts [2]. There are many positive health-related actions offered by this kind of food, including the potential to boost the immune system, reduce the risk of cardiovascular problems, osteoporosis, obesity, and some types of cancer, as well as improve memory and physical condition [3]. As consequence, the functional food industry is characterized by dynamic growth, and producers are responding actively by supplying new products that meet consumers’ needs. Innovation in the area of functional food products is very intense and desirable.

Honey is a natural food product produced by bees from the nectar of flowers or honeydew, and has been used since ancient times as part of traditional medicine as an antibacterial, antioxidant, antitumor, anti-inflammatory, and antiviral agent. Lately, as with many foods, honey has been enriched with functional ingredients. Some herbs, vegetables, and fruits have been trialed for this purpose [4,5,6]. A variety of quite new and interesting products has appeared on the Polish market, especially creamed honey with the addition of dried herbs which have been rarely studied [7]. In our previous studies, we used chokeberry (*Aronia melanocarpa*) fruits [8] and *Morus alba* fruits and leaves [9] for rape honey enrichment. We found that the introduction of various plant additives to honey creates different health-promoting properties of the final product, depending on the pharmacological properties of the plant. Although the synergistic effect of honey and herbs is always expected, it should be confirmed in any case. The additional benefit of such combinations is greater consumer acceptability due to the masking of the bitter taste of the herbs by the sweet honey matrix [6,7,8,9].

Due to increasing awareness about optimal nutrition among customers, the global consumption of fruits and fruit-based products has increased considerably. On the other hand, therapeutic efficacies of fruit extracts include immune-modulatory properties and influence on the immune system of the human body; therefore, health care advisors and nutrition counselors recommend the inclusion of fruits and fruit-based products in our diets [10].

Some fruits known as “superfruits” possess especially high pro healthy properties; *Rubus*, one of a hundred genera in the family Rosaceae, is one such “superfruit”. There are 250 species of *Rubus*, especially present in the northern temperate zone, with the majority being indigenous to Europe. The best-known *Rubus* species are *R. idaeus* (raspberry) and *R. fruticosus* (blackberry). However, the name *R. fruticosus* does not refer to a single species but is used as collective species name, comprising about 2000 described European species [11]. *Rubus* fruits are known for their delicious taste, pleasant flavor, and nutritional profile, and are consumed fresh or processed (dried, frozen, pureed and freeze-drying lately) as ingredients of many dishes—jams, ice cream, desserts, bakery products, salads, and drinks, whereas leaves, whether dried or fresh, are used as a tea [12]. Raspberry and blackberry species are known to exhibit a wide range of pharmacological activities and they have long been traditionally applied for their antiseptic, antimicrobial, cardioprotective, and antioxidant properties [11,13]. Although *Rubus* fruits are well known and widely consumed, the leaves of these plants, classified as herbal raw materials, are rarely consumed and only in the form of infusions. *Rubus* plants in Poland are grown on a large scale; we are at the forefront of exporters to external markets [14]. Thus, broadening the use of leaves would increase the economic efficiency of cultivation. New designed products based on honey and *Rubus* leaves will enrich the assortment of functional foods and will allow the leaves to be introduced into the diet in a convenient and organoleptically attractive form.

Given the facts of the efficacy of edible fruits and honey, we decided to use blackberries and raspberries for rape honey enrichment. Unfortunately, there is no data in the literature describing the chemical composition and health-promoting properties of such new functional apiphytotherapeutic food. Thus, the aim was to obtain the new functional products: honey with *Rubus* spp. fruits, and honey with *Rubus* spp. leaves, and to test the effect of such combinations against viruses and bacteria. Knowing that raspberries and blackberries, although they belong to the same Rubus genus, differ in chemical composition and biological activity, we decided to check the resultant effect of their combination with honey. Moreover, the stability of enriched honey during storage was evaluated for the first time. The hypothesis that designed novel products will have enhanced pro-health properties and anti-coronaviruses activity, as a consequence of the synergistic effect between honey and plant additives, was verified.

## 2. Results

By introducing plant additives during honey creaming, innovative products were obtained, characterized by an attractive color (pink-red for fruit, and green for leaves), and the intensity of which increased with increasing share of the additive in honey (Figure S1). Creamy-textured products with visible plant particles were obtained.

### 2.1. The Comparison of Antioxidant Properties of Studied Rubus *spp.*

The antioxidant potential of fruits and leaves of *Rubus fruticosus* (blackberry) and *Rubus idaeus* (raspberry) was compared taking into account the origin of samples: commercial (C) and harvested from the crop (H) (Table 1). Compared to “superfruits” previously used to enrich honey, *Rubus* spp. fruits showed a slightly lower content of total polyphenols (for mulberry up to 111 mg GAE/g and 55–59 mg GAE/g for chokeberry fruits) [8,9], whereas the values for leaves were comparable for these obtained for *Morus* sp. leaves (57–76 mg GAE/g) [9]. The comparison was possible thanks to the use of the same analytical protocol.

*Rubus* sp. leaves were characterized by a higher content of total phenols and flavonoids compared to fruits. Furthermore, those from organic farming were more abundant in bioactive compounds than those available commercially. The difference in favor of the BH and RH samples was about two-fold. In the case of fruit, no significant differences were observed between the counterparts from the two sources. Flavonoids constituted a small percentage (up to 26% in the case of leaves and below 1.5% in the case of fruits) of the total content of phenolic compounds, which results from the predominant content of phenolic acids and ellagitannins in various *Rubus* species [15,16]. Varied data have been reported in the literature: the content of polyphenols in *Rubus* leaves ranged from 0.3 to 2.2 mg GAE/g dry mass in raspberry leaves [17] and from 0.25 to 0.35 mg GAE/g d.m. [18] up to 84.64–144.20 mg GAE/g d.m. [19] in the case of various varieties of blackberry. The content of phenols in fruits was more stable and ranged from 22.3 to 26.85 mg GAE/g d.m. for dried blackberries [20] and 26.31–38.51 mg GAE/g d.m. for dried raspberries [21]. The content of flavonoids in *R. fruticosus* leaves at a mean level of 17.49 mg QE/g d.m. was reported by Ziemlewska et al. (2021) [22]. The presented data are in line with the results found in our study.

An important group of polyphenols present in fruits of the *Rubus* genus is anthocyanins. According to our research, blackberries contain about 50% higher content compared with raspberries, and a slightly higher content of anthocyanins was recorded for commercially available raspberry fruit. It is known that blackberries contain much higher levels of anthocyanins than raspberries, which is reflected in the color of the fruit [14,23]. Moreover, the values obtained for raspberry fruit are consistent with the data presented in the literature. Stamenkovic et al. (2019) detailed the content of anthocyanins per g of dried fruit as being from 2.15 to 2.48 mg and for freeze-dried fruit from 2.05 to 2.63 mg, depending on drying conditions [24]. Other sources report higher anthocyanin content in raspberries: i.e., from 5.71 to 13.8 mg Cy-3-glu/g d.m. [25].

The antioxidant activity was measured by two methods: FRAP and DPPH. Significantly higher values were obtained for leaves, irrespective of the species. As in the case of the content of phenolic compounds, the advantage of raw materials from organic farming was observed. The obtained results were strongly correlated, and the calculated values of the Pearson correlation coefficient ranged from 0.7 for TFC vs. FRAP to 0.98 for TPC vs. DPPH. Raspberry, blackberry and other *Rubus* sp. fruits are recognized sources of antioxidants. The leaves of these plants are slightly less known for this property, although they have been studied around the world. The high antioxidant potential of leaves of many *Rubus* species has been confirmed by ABTS and FRAP methods [15]. In the study by Buricova et al. (2011) [26], it was found using the DPPH method that raspberry leaves were active at the level of 105.2 mg AAE/g, whereas blackberry leaves were stronger—152 mg AAE/g. Raspberry leaves from conventional and organic cultivation were compared in terms of antioxidant capacity using the ABTS method, and it was found that organic farming contributes to an increase in the antiradical effect (61.78 and 72.93 mmol TE/100 g, respectively) [27]. Moreover, varietal differentiation was also demonstrated, from 58.27 to 88.10 mmol TE/100 g d. w. of leaves, which are higher values than those obtained in this study. Strongly higher values for blackberry fruits were also obtained by Lutz et al. (2015) [28], who determined the ability to reduce the DPPH radical at the level of 1203.8 µmol TE/g d.m. However, it should be remembered that such factors as the plant variety, climatic conditions, and agrotechnical procedures, as well as the method of preparing the extract for analysis, have a huge impact on the antioxidant properties.

### 2.2. The Effect of Rubus *spp.* Addition on Antioxidant Properties of Honey

#### 2.2.1. Antioxidant Activity and Polyphenols

The same parameters as for plant extracts were analyzed for creamed honeys with the addition of previously tested fruits and leaves of two *Rubus* species. The results obtained after monthly storage of honeys are summarized in Table 2. A significant increase in all tested parameters for enriched honeys was noted compared with the raw rape honey (control sample) (*p* < 0.05).

In the basic rape honey, the total content of phenolic compounds was determined at the level of 355.65 mg GAE/kg which is a typical value for this variety of Polish honey [29,30,31]. Among nectar honeys, rape honey is the variety with the lowest levels of polyphenolic compounds and antioxidant activity. In our previous studies, for this variety TPC, on average, 254.52 mg GAE/kg was found [32]. The flavonoid fraction of total polyphenols was 91.6 mg QE/kg, which is 25.75% of total polyphenols.

The addition of *Rubus* sp. leaves and fruits in all the variants used resulted in a significant increase in the content of polyphenols as well as anti-radical activity and reducing ability. There is a clear relationship between the dose of the applied additive and the enrichment of the product with bioactive compounds. The increase in the content of phenolic compounds is even over 400% compared to the control honey, with the addition of 4% fruit or 1% leaves of *Rubus* sp. The enhancement of antioxidant properties resulting from the introduction of fruit or herbs to honey is greater than it would appear from simple summation. We explain this phenomenon by hyperadditional synergism, which means that the action of two preparations used together is stronger than the sum of their single actions. Similar effects were previously observed when enriching rape honey with mulberry leaves and fruit [9] and chokeberry fruit [8]. Another study showed the synergism of the antioxidant and scavenging effects of honey and the fruit of *Rosa* spp. [33]. An important effect, especially due to the organoleptic properties of the product, i.e., color and taste, is the introduction of fruit anthocyanins into the honey, but their content was unfortunately not determined in the final product. The correlation analysis of the results obtained for creamed honeys is presented in Table 3.

The values of Pearson’s coefficients indicate quite strong correlations, especially of the total phenolic content, with the antioxidant activity of the samples. This confirms the significant influence of the addition of *Rubus* sp. fruits and leaves on the health-promoting properties of the final products obtained.

**Figure 1:**
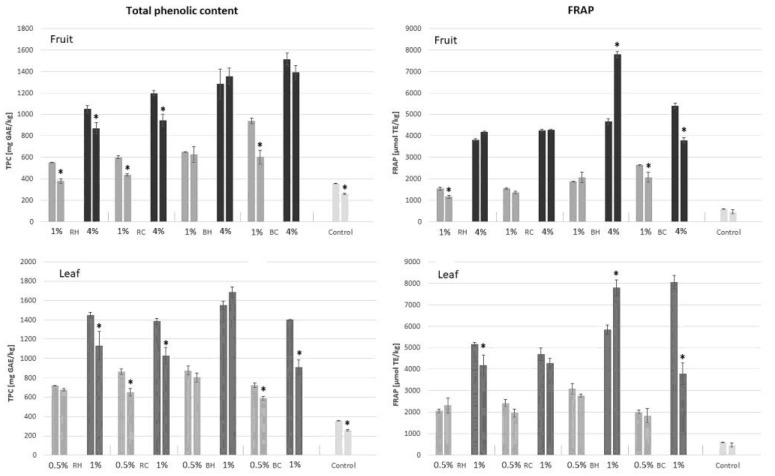
Changes in TPC and FRAP values of enriched honey after 90 days of storage under room conditions. *—means differ significantly (*t* test, *p* = 0.05).

Two parameters (TPC and FRAP) of creamed honey stored for 90 days in room conditions were determined to assess obtained product stability. The results are shown separately for the honey enriched with leaves and fruits in Figure 1.

In most cases, including the control sample, a decrease in the content of phenolic compounds and the reducing power of FRAP was observed. Only in a few cases (mainly with the addition of organic blackberry fruit and leaves (BH) in a higher proportion), the results indicate an increase in these product parameters. However, the observed significant changes were in the range of 3–30% in most cases.

During long storage of honey, significant decreases in antioxidant activity were previously observed, up to over 60% after 12 months [34]. Significant drops in antioxidant activity, as well as the total content of phenols and flavonoids, were previously observed also for acacia honey and multifloral honey [35]. For acacia honey, a different trend was reported, an initial increase in the content of flavonoids and their gradual loss after 6 months of storage [35]. In turn, Monggudal et al. (2018) [36] observed a significant increase in the TPC value for several tested honeys. However, in the available literature, there was no presented clear explanation of the mechanisms that influence tested parameters during long-term storage.

#### 2.2.2. HPTLC Polyphenolic Profile

**Figure 2:**
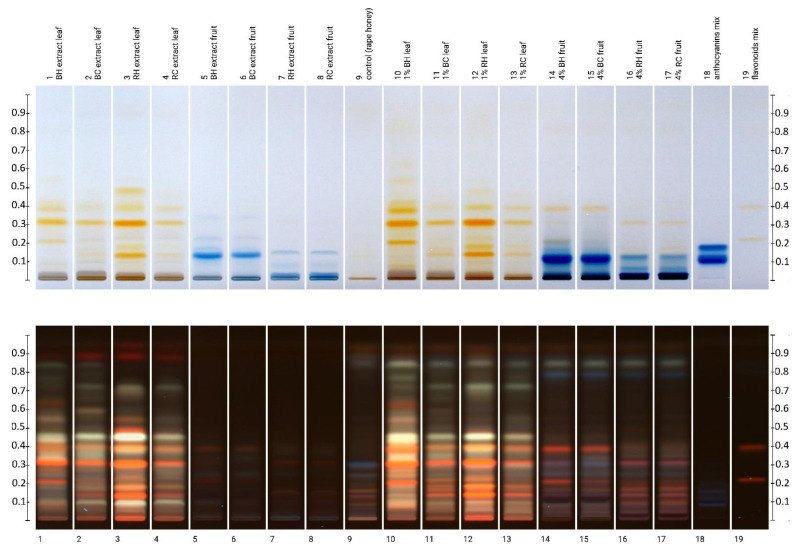
HPTLC plate images after NP-reagent derivatization: in visible light (**top**) and in UV 366 nm (**bottom**). Anthocyanins standards mix (track 19): cyanidin-3- galactoside, cyanidin-3-glucoside, and cyanidin-3-arabinoside; flavonoids mix: rutin, hyperoside, (+)-catechin (in order of increasing Rf values).

The polyphenol profiles of raspberry and blackberry (fruit and leaf) extracts, as well as creamed honey with the addition of these *Rubus* materials, were qualitatively compared using high-performance thin layer chromatography (HPTLC) with NP-reagent derivatization. Chromatograms after visualization in visible and UV light are presented in Figure 2.

Both chromatograms of leaf extracts and honey produced with the addition of leaves of both *Rubus* species show numerous yellow bands in visible light and orange in UV light. They correspond to the flavonoids dominating in the leaves, among which, by comparison with the standards, it was possible to identify two quercetin glycosides: rutin (Rf = 0.22) and hyperoside (Rf = 0.40). The (+)-catechin band (Rf = 0.80), gray in UV light, is less visible, its presence has not been confirmed, and perhaps it is covered by other, more abundant compounds. The remaining intense yellow bands may result from other quercetin or kaempferol glycosides, which compounds are the dominant representatives of the flavonoids in raspberry and blackberry leaves [15,19,37]. In all leaf extracts, as well as in honeys with their addition, the band at Rf = 0.33 dominates, which may correspond to isoquercitrin (quercetin-3-glucoside), which, according to the literature, is the main quercetin glycoside of *Rubus* sp. leaves [15,27]. In addition, numerous white and gray UV bands were found, most likely from numerous phenolic acids, whereas red bands with high Rf values were attributed to chlorophylls. Blackberry and raspberry leaves contain many phenolic acids, especially caffeic acid, and its derivatives, as well as ellagic, chlorogenic, and p-coumaric acids [15,19,37].

In the case of fruit extracts, anthocyanins were detected: cyanidin-3-galactoside (Rf = 0.11) as dominant in blackberry fruits and cyanidin-3-glucoside (Rf = 0.13) in raspberries. In addition, the bands from other anthocyanins (e.g., at Rf = 0.06 in raspberry samples), which was not identified due to a lack of standards. Cyanidin-3-arabinoside (Rf = 0.18) was not found in tested extracts. Numerous anthocyanins have been identified in raspberry fruits, mainly cyanidin derivatives (sophoroside, glucoside, rutinoside as well as diglucosides) and pelargonidin [38,39,40]. Blackberries are also dominated by cyanidin glycosides [41,42,43,44].

Honey enriched with the addition of *Rubus* sp. leaves and fruits showed a similar polyphenol profile as the additives used. Compared with the control rape honey, their profiles were much richer in multi-colored bands of phytochemicals originating from plant additives. At the same time, new bands appear in the profiles of honey with the addition of fruit, e.g., intensively blue In UV light at Rf = 0.78, not present in the control honey or fruit extracts. This may suggest probable interactions between the honey and fruit components and the likely formation of new compounds or complexes.

Comparing the profiles between organic and commercially available raw materials is in favor of the former, especially in the case of leaves. The intensity of the bands in the chromatograms of these samples is much higher, which indicates a higher content of metabolites in the extracts. Additional bands are also present in them, e.g., in the BH sample, there is an intense orange band at Rf = 0.63, which is absent in the commercial counterpart. This is confirmed by the data given above for the total content of polyphenols and flavonoids in these samples. Higher enrichment is also visible in honeys with the addition of leaves from organic farming. A similar effect of enriching honey with polyphenolic compounds, including anthocyanins, was previously observed for honeys creamed with chokeberry fruit [6]. The pattern of the bands of honey extracts with the addition of raspberry and blackberry fruit is more intense than in the case of the extracts themselves, which can be explained by the concentration of the sample as a result of solid-phase extraction at the stage of preparation for HPTLC analyzes.

### 2.3. HPLC Analysis

**Figure 3:**
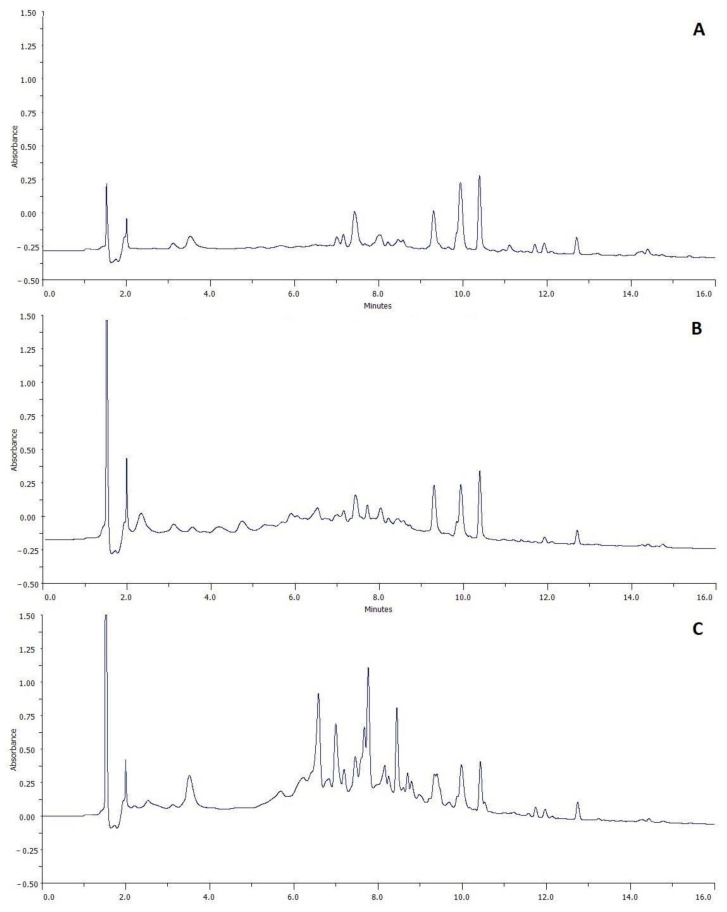
HPLC-DAD chromatograms for control honey (**A**), honey enriched with *R. idaeus* fruits (**B**) and leaves (**C**).

Enrichment of honey with polyphenols due to the addition of Rubus spp. fruits and leaves was confirmed by HPLC-DAD chromatographic analysis (Figure 3).

Compared with the starting honey (control, Figure 3A), which contains a few phytochemicals (p-coumaric acid, ferulic acid, benzoic acid, kaempferol, pinobanksin, pinocembrin), honey creamed with Rubus additives contain numerous compounds from several classes of polyphenols. The addition of fruit introduces mainly anthocyanins (cyanidin derivatives) and procyanidin tannins (Figure 3B, Table S1). This was the expected effect as raspberry and blackberry fruits are a rich source of these compounds [38,39,44]. The introduction of leaves to honey results in greater enrichment in polyphenols, including numerous derivatives of flavonoids (mainly kaempferol), as well as procyanidins (Figure 3C, Table S1). The presence of these compounds has been previously confirmed by chromatographic analyzes in numerous studies [15,19,27].

### 2.4. Antiviral Potential of Honey Enriched with Rubus *spp.*

Viral infections are known as one group of the major causes of death worldwide and affect three to five million patients annually; however, relatively few antiviral drugs are now available, and vaccination is limited to only a few [45,46]. There are 219 virus species that are known to be able to infect humans, and more than two-thirds of human viruses can also infect non-human hosts, mainly mammals, and sometimes birds [47]. The COVID-19 pandemic, which is caused by coronavirus SARS-CoV-2, is the main reason for growing interest in searching for antiviral agents. While commonly used antivirals often show limited efficacy and serious adverse effects, herbal extracts have been in use for medicinal purposes since ancient times and are known for their antiviral properties and more tolerable side effects [46]. Among many natural substances widely used in traditional medicine for fighting against bacterial and viral diseases, honey and plants such as raspberries and blackberries can be mentioned [11,48,49,50].

Due to the fact that working with highly infectious human coronaviruses requires laboratories with biosafety levels, more than three-quarters of researchers have valued the potential of bacteriophages (bacterial viruses, phages) as an appropriate viral surrogate to measure human enveloped virus’ survival, transfer, and removal. Phages are very specific and only attack selected hosts, do not require specialized biocontainment precautions, are relatively easy to produce within a laboratory, and are safe for humans. Bacteriophages represent good surrogates for the study of airborne viruses and display structural features similar to many eukaryotic viruses [11,51]. Phage phi6 is a segmented RNA virus that possesses a phospholipid envelope with spike proteins at its surface. Because of its structure, it is similar to several human viruses such as the influenza virus, SARS-CoV-1, SARS-CoV-2, and MERS-CoV, and is useful as a coronavirus surrogate to assess the effectiveness of anti-SARS-CoV-2 approaches, providing important insights concerning the COVID-19 pandemic and human public health [11,50,51,52,53,54]. That is why bacteriophage phi6 was used in our experiments.

**Figure 4:**
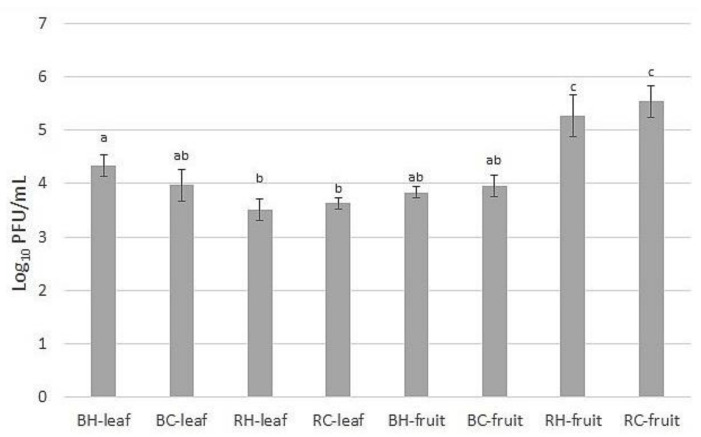
Reduction in viral particles after 10 min. of incubation with blackberry (BH—harvested; BC—commercial) and raspberry (RH—harvested; RC—commercial) fruits and leaves extracts, dissolved in 5% DMSO at a concentration of 5 mg/mL. ^a,b,c^—means sharing the same superscript letter in a column do not differ significantly (Tukey’s test, *p* = 0.05).

Antiviral potential of crude extracts of tested *Rubus* species was compared and presented in Figure 4. The highest antiviral potential was observed for raspberry fruit extracts (>5 log_10_ PFU/mL reduction) and for blackberry leaf extracts (around 4 log_10_ PFU/mL reduction). The lowest viral particles reduction was shown for raspberry leaf extracts. The differences between the source of plant material (H or C) were not significant.

**Figure 5:**
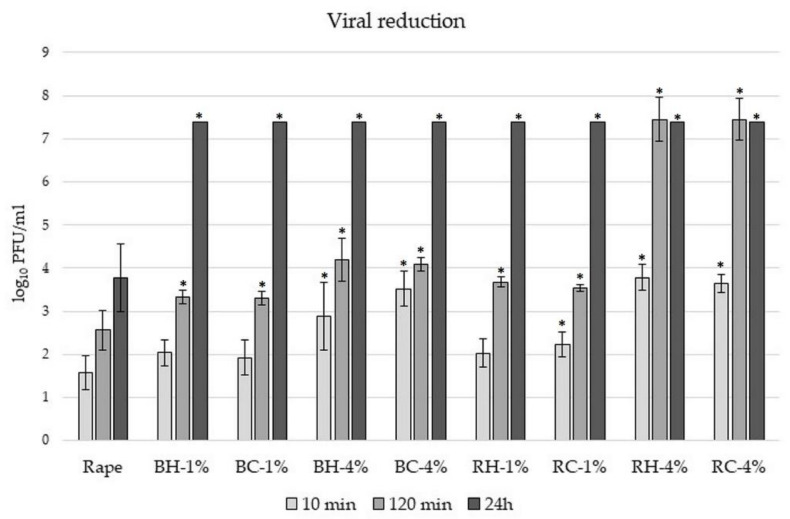
Reduction in viral particles after different times of incubation with 25% (*w*/*v*) water honey samples enriched with *Rubus* fruits. Rape—rape honey control, BH—honey with harvested blackberry, BC—honey with commercial blackberry, RH—honey with harvested raspberry, RC—honey with commercial raspberry; 1–4%—concentration of additive. *—means differ significantly (*t* test, *p* = 0.05).

Antiviral activity of honey enriched with *Rubus* spp. fruits and leaves was determined in water solutions of honey samples (25%, *w*/*v*) after a prolonged contact time: 10 min, 2 h, and 24 h, and the remaining infectivity was determined by a double agar overlay plaque assay [8]. In the control sample, the bacteriophage was incubated with STM buffer. As we can see in Figure 5, the viral activity was reduced by around 1.5 log_10_ PFU/mL after 10 min of incubation for the rape honey control.

Viral reduction slightly increased in samples with 1% of fruits addition (reduction around 2 log_10_ PFU/mL). The differences between the source of fruits (H and C) were insignificant and below 10%. Similarly, in our previous paper, we have shown that the addition of 1% of chokeberry fruits did not significantly influence honey’s antiviral potential in comparison with rape honey [8]. The longer the incubation time, the higher the antiviral potential of honey enriched with fruits, with the highest observed for honey with raspberry fruits (the viral reduction at least 7.4 log_10_ PFU/mL after 2 h of incubation). These results confirm the data obtained for pure fruit extracts, in which raspberry fruit extracts showed the strongest antiviral potential (Figure 4). It can be observed that the addition of *Rubus* fruits at a concentration of 4% increases its antiviral potential, by around twice after 10 min of incubation in comparison with rape honey, and totally inhibited viral particles after 24 h of incubation (viral reduction at least 7.4 log_10_ PFU/mL).

However, there is no correlation between antioxidant potential, phenolic content, and antiviral properties of honey enriched with raspberry and blackberry fruits. Probably antiviral potential of *Rubus* fruits depends not directly on total phenolic content or antioxidant potential, but on the content of some specific phenolic compounds, such as different classes of flavonoids, etc., which concentration is different in blackberry and raspberry fruits [55,56]. Danaher et al. (2011) [57] reported that blackberry fruit juice extract inhibits and inactivates *Herpes simplex* virus type 1 (HSV-1) replication in oral epithelial cells by 99%, mainly due to the presence of rich amounts of anthocyanins and ellagitannins. These proprieties depend strongly on the composition of the berry tested, and on the type of virus targeted. The variety of phytochemicals in berries makes them very interesting as sources of antiviral compounds [58]. On the other hand, honey enriched with higher content of *Rubus* fruits (4%) exhibited 3–4 times higher antioxidant potential and phenolic content, which is also observed in the greater antiviral potential of these honey samples.

**Figure 6:**
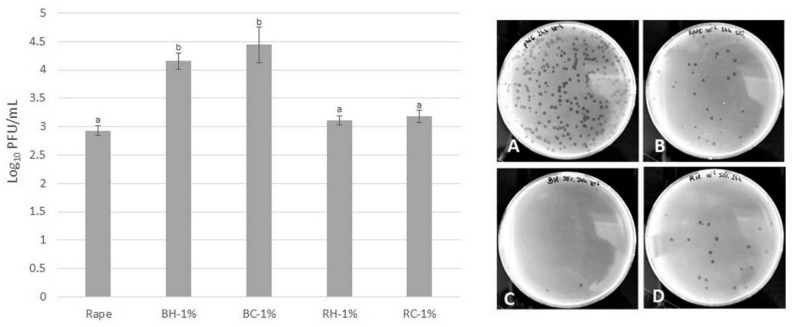
Reduction in viral particles after 24 h of incubation with honey enriched with *Rubus* leaves (50% *w*/*v* water honey samples) (on the left). Plaques formed by phi6 in the double agar overlay method after 24 h of virus incubation with 50% (*w*/*v*) water honey samples—(**A**)—control phage phi6 in STM buffer, sample dilution 10^−4^, (**B**)—rape honey, sample dilution 10^−2^, (**C**)—honey with 1% blackberry leaves extract (BH), sample dilution 10^−2^ (**D**)—honey with 1% raspberry leaves extract (RH), sample dilution 10^−2^ (on the right). ^a,b^—means sharing the same superscript letter in a column do not differ significantly (Tukey’s test, *p* = 0.05).

In the case of the addition of *Rubus* leaves to the concentration tested (0.5 and 1%), no influence on antiviral potential of 25% honey after 24 h of incubation was observed (data not shown). For this reason, the antiviral potential of honey enriched with leaf extracts was analyzed for 50% of honey samples enriched with 1% of leaves extracts after 24 h of incubation. The results are present in Figure 6. A higher antiviral potential was observed for honey enriched with blackberry leaves (>1 log reduction in PFU/mL), whereas raspberry leaves addition did not significantly change the antiviral potential in comparison with control rape honey.

### 2.5. Antibacterial Properties of Rubus *spp.*-Enriched Honeys

**Figure 7:**
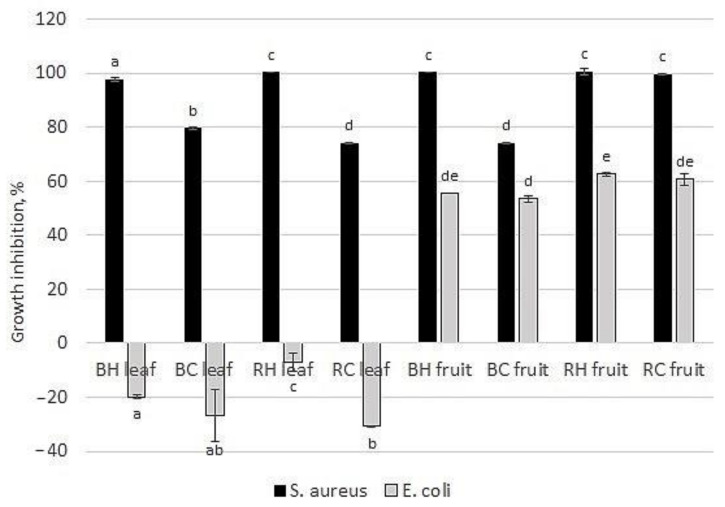
Bacterial growth inhibition of blackberry and raspberry leaves and fruits at a concentration of 10 mg/mL. BH—harvested blackberry, BC—commercial blackberry, RH—harvested raspberry, RC—commercial raspberry. ^a,b,c,d,e^—means sharing the same superscript letter in a column do not differ significantly (Tukey’s test, *p* = 0.05).

The antibacterial potential of crude extracts of *Rubus* fruits and leaves is shown in Figure 7. As can be seen, higher antibacterial potential was observed against *S. aureus* than against *E. coli*. Stronger bacterial growth inhibition was observed for fruit extracts than for leaf extracts. Surprisingly, at the analyzed concentration leaves extracts of blackberry and raspberry supported bacterial growth. Similarly, De Santis et al. (2022) [49] did not observe any inhibitory effect of raspberry leaf extract against *E. coli* determined by the well diffusion method. On the other hand, Welia et al. (2020) [59] demonstrated that the antibacterial potential of methanolic extract from blackberry leaves (*Rubus fruticosus*) did not inhibit the growth of *S. aureus* (at concentrations 250–2000 µg/mL) determined by the agar well diffusion method but and inhibited *E. coli* growth. However, the inhibition was observed only for lower concentrations (250, 500 µg/mL) at comparable levels (diameter of inhibition zone—6 mm), whereas the highest used dose (1000 µg/mL) was not effective. The differences in antibacterial potential shown by different authors may be due to the procedure of extraction, type of solvent used, geographical origin of plants or time of leaves harvesting. Similarly, in our previous work, the strong anti-staphylococcal activity of blackberry (*R. plicatus*) and raspberry *(R. idaeus*) fruit and leaf extracts was demonstrated [60].

Our results on the antibacterial potential of analyzed plants are in accordance with their antioxidant potential and total phenolic content. Anti-staphylococcal properties of *Rubus* leaves are stronger for leaves collected from the local harvest, with around twice higher antioxidant potential and phenolic content, than extracts prepared from commercial plant material.

**Figure 8:**
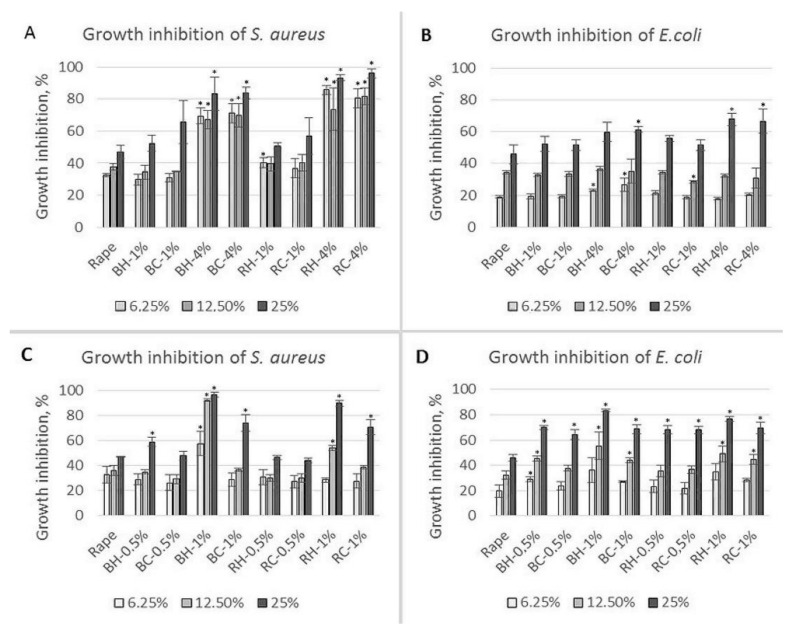
Bacterial growth inhibition at different honey concentrations (6.25%, 12.5%, and 25%) of the control (rape) and enriched honey. (**A**,**B**)—honey enriched with fruits, (**C**,**D**)—honey enriched with leaves. Rape—rape honey control, BH—honey with harvested blackberry, BC—honey with commercial blackberry, RH—honey with harvested raspberry, RC—honey with commercial raspberry. 0.5–4%—concentration of additive. *—means differ significantly (*t* test, *p* = 0.05).

As could be expected, *Rubus*-enriched honey showed antibacterial potential against *S. aureus* and *E. coli*. The results, presented in Figure 8, show a percentage of bacterial growth inhibition in comparison with the control sample (bacterial growth without honey addition). Generally, rape honey, produced in large amounts in Poland, and used in this study, possesses the weakest antibacterial potential among other honey types [61,62]. As can be seen in Figure 8A, rape honey and honey with 1% addition of berry fruits, at a concentration of 6.25%, inhibited *S. aureus* growth by 30%, whereas *E. coli* growth was inhibited only by around 20% (Figure 8B). The antibacterial effect was dose-dependent, and with increasing concentration of berry fruit additives the antibacterial potential against *S. aureus* of samples enriched with 4% of berries was even observed at 6.25% honey concentration; growth reduction of 70% was observed for blackberries and 80–85% for raspberries. A weaker effect was observed for *E. coli* growth inhibition where similar values of inhibition of around 60% for blackberry addition and around 67% for raspberry fruits addition were as observed for the higher concentration of honey (25%) enriched with 4% of fruits. Generally, there were no statistically important differences between the two sources of berry used. Comparing the results with antioxidant and phenolic content, it can be seen that in most cases, differences between the two berry sources (similar blackberry and raspberry) ranged from 0 to a maximum 20% (with exceptions for TPC and FRAP for honey with 1% of blackberry addition and TFC for honey with 4% of raspberry addition). However, *S. aureus* was more sensitive to analyzed honey than *E. coli*.

### 2.6. The Effect on Biofilm Formation

Biofilm formation is a self-protective mechanism where bacteria aggregate to create a complex structure to resist harsh conditions. It causes an increase in bacterial pathogenicity, higher tolerance to conventional antimicrobial agents, and resistance to phagocytosis. As a result, microorganisms become more difficult to eradicate from living hosts. In clinical samples, staphylococci that are responsible for infections often exist in the form of biofilms [63].

The efficacy of honey enriched with berries in the prevention of biofilm formation was measured using a static microtiter plate biofilm assay against *S. aureus*.

**Figure 9:**
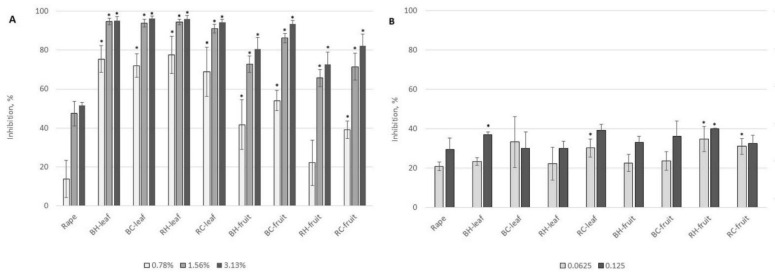
Effect of different concentrations of *Rubus*-enriched honey on *S. aureus* biofilm formation (**A**) and elimination of pre-established biofilms (**B**)—1% of leaves addition, 4% of fruits addition. Rape—rape honey-control, BH—honey with harvested blackberry, BC—honey with commercial blackberry, RH—honey with harvested raspberry, RC—honey with commercial raspberry. *—means differ significantly (*t* test, *p* = 0.05).

The results shown in Figure 9 confirmed that honey enriched with berry fruits and leaves was effective at preventing *S. aureus* biofilm formation. However, the addition of honey had a very small impact on the dispersion of an established biofilm (Figure 9B), even at lower concentrations of honey-supported biofilm growth (data not shown). Grecka et al. (2018) [63] also observed that even in the most active honey, such as Manuka honey 550, inhibition of biofilm formation requires at least 25% honey concentration. Additionally, for Polish honey samples, complete eradication of biofilm was achieved at the 50% honey concentration. The effect of enriched honey in inhibiting biofilm formation was more pronounced than that in eliminating preformed biofilms. Effectiveness in antibiofilm formation may be partially dependent on the inhibition of bacterial growth in the presence of honey. However, honey enriched with 4% of *Rubus* fruits inhibited *S. aureus* growth at 25% concentration (MIC 90), whereas inhibition of biofilm formation was observed for lower concentrations of honey samples (3.13%—biofilm inhibition at 80–90%). The results show that the honey samples exhibited a remarkable capacity to inhibit the formation of the bacterial biofilm. Nazarro et al. (2020) [64] reported that some monofloral honeys exhibited a remarkable capacity to inhibit the formation of *S. aureus* biofilm. They show that *S. aureus* was more sensitive at a metabolic level (61.63% inhibition in the presence of the tree of heaven honey). Shirlaw et al. (2020) [65] reported that heater honey at a concentration of 0.25 mg/mL inhibited biofilm formation in *S. aureus* (69.6%).

## 3. Materials and Methods

### 3.1. Reagents

Most of the chemicals and reagents were obtained from Sigma Aldrich (Saint Louis, MO, USA), and buffer components were purchased from POCH (POCH, Gliwice, Poland). Media for antibacterial and antiviral potential were purchased from BTL, Poland (Mueller Hinton Broth—MHB) and Biomaxima, Poland (Tryptic Soy Broth—TSB, Tryptic Soy Agar—TSA, bacteriological agar).

### 3.2. Honey and Fruit Samples

Two samples of fruits and leaves of blackberries (*Rubus fruticosus*) (B) and raspberries (*Rubus idaeus*) (R) were used. One of them was commercially available, bought in a local health store in dried form (BC and RC), the second was collected at the optimum ripening time in 2020, from crops located in south-eastern Poland (BH 50.27 N, 21.37 E and RH- 50.47 N, 23.62 E). Fresh leaves were dried at room temperature in the dark and fruits were lyophilized (Alpha 1–2 LD plus, Martin Christ, Osterode am Harz, Germany). Dried materials were ground into a coarse powder with a laboratory mill (MK-06M, MPM, Milanówek, Poland).

Rape honey was obtained from the ecological apiary localized in the Podkarpackie region (50.31 N, 21.28 E) in the 2020 beekeeping season. Before creaming, the honey was completely liquefied at 45 °C for 48 h in a laboratory dryer (SLN 53 STD, Pol Eko, Wodzisław Śląski, Poland).

### 3.3. Preparation of Rubus-Enriched Honeys

Enhancement of rape honey with fruits and leaves was carried out as detailed by Tomczyk et al. (2019) [9]. To start the process of crystallization, liquefied honey was inoculated with crystalized honey (99:1 g) and mixed for 60 s four times a day. Then, powdered blackberry and raspberry leaves were introduced to honey in amounts of 0.5% and 1% (*w*/*w*), whereas fruits were in concentrations 1% and 4%. Samples were mixed again for 60 s, then stored at 4 °C for 3 days and mixed twice a day. Each variant was prepared in two technical replications, which finally resulted in 32 test samples. Rape honey was used as a control. Over the next 30 days, honeys were stored for crystallization completed at room temperature (20 ± 2 °C) in the dark and subjected to analysis. Selected parameters were checked again after 90 days of storage.

### 3.4. Sample Preparation for Analysis

Powdered leaves and fruits (2.5 g) were extracted with 25 mL aqueous ethanol (50%) using an ultrasound-assisted method (700 W, 40 kHz; SONIC-10, Polsonic, Warszawa, Poland). The extracts were filtered through a paper filter and stored in a refrigerator until analysis. For antibacterial and antiviral properties, extracts were lyophilized (Alpha 1–2 LD plus, Martin Christ, Osterode am Harz, Germany) and dry mass was suspended in 5% DMSO (dimethyl sulphoxide) to an appropriate concentration, then sterilized by syringe filters (0.22 µm).

Honey extracts for all analyses were prepared as follows: 0.5 g of honey was dissolved in 10 mL of distilled water, obtaining 5% solutions. For chromatographic analysis only, honey samples were prepared by solid-phase extraction using C18 SepPak cartridges (Waters, Milford, MA, USA). Briefly, 20 g of each honey was dissolved in 100 mL of acidified water (pH = 2) and passed through the cartridge preconditioned with methanol (10 mL) and acidified water (10 mL). Sugars were removed by acidified water elution, and the polyphenolic extract was eluted with methanol (2.5 mL).

### 3.5. Total Polyphenol Content (TPC)

Total phenolic content was measured using the Folin–Ciocalteu colorimetric method detailed by Dżugan et al. (2018) [32] with minor modifications. Appropriate dilutions of honey samples or plant extracts in the volume of 20 µL were mixed with 100 µL of 10% Folin–Ciocalteu reagent and 80 µL of 7.5% of sodium carbonate in microplate wells. After 60 min of incubation, the absorbance of the samples was measured at 760 nm, against a blank, using an Epoch-2 microplate reader (BioTek, Winooski, VT, USA). The results were expressed as mg of gallic acid equivalents (GAE) per g of the dry mass sample.

### 3.6. Total Flavonoids Content (TFC)

The content of flavonoids was assessed using the method of Biju et al. (2015) [66]. For this purpose, 100 µL of properly diluted honey samples or plant extracts were mixed with 100 µL of 2% AlCl_3_ (in methanol). Samples were incubated for 10 min at room temperature, then absorbance at 415 nm was measured with an EPOCH 2 microplate reader against methanol as a blank. The results (TFC) were expressed in mg of quercetin equivalent (QE) per 1 g of dry mass.

### 3.7. Total Anthocyanins Content (TAC)

The total anthocyanins content in *Rubus* fruits was determined by the pH differential method according to Giusti and Wrolstad (2001) [67]. The extracts were appropriately diluted with two buffers: 0.025 M potassium chloride buffer of pH 1, and 0.4 M acetate buffer of pH 4.5. After 15 min incubation at room temperature, the absorbance was measured against distilled water at 520 and 700 nm (Biomate 3 spectrophotometer (Thermo Scientific, Waltham, MA, USA). The absorbance of each sample was calculated using the following Formula (1):

A = (A520 − A700) _pH1.0_ − (A520 − A700) _pH4.5_ (1)

The total anthocyanin content expressed as cyanidin-3-glucoside equivalents in mg per one gram of fruits was calculated using the following Formula (2):

TAC = (A × MW × DF × 1000)/(ε × 1)(2)

where: A—calculated absorbance; MW—molecular weight (449.2 g/mol for cyanidin 3-glucoside); DF—dilution factor (20); ε—molar absorptivity of cyanidin 3-glucoside (26,900 dm^3^/mol × cm)

### 3.8. HPTLC Analysis—Polyphenolic Profile

Polyphenol profiles were obtained by the HPTLC method, using the Camag System (CAMAG, Muttenz, Switzerland), consisting of an automatic applicator (Linomat 5), a developing chamber (ADC2), a derivatizer and a visualization apparatus (TLC Visualizer 2). Chromatographic separation was performed on silica gel 60 F254 HPTLC plates (glass plates 20 × 10 cm), purchased from Merck (Darmstadt, Germany), with mobile phase system ethyl acetate:water:formic acid:acetic acid (15:2:1:1). The samples were applied at 6 µL for additive extracts, 5 µL for honey extracts, and 2 µL for standards, as 6.5 mm bands, 8 mm from the edge of the plate. The plate was developed to 70 mm in a saturated chamber. The chromatographic images were digitally processed using specialized HPTLC software (visionCATS, CAMAG). The plate was derivatized with Natural Product reagent/PEG 400 and re-analyzed in UV (366 nm) and visible light.

### 3.9. HPLC-DAD Analysis

The HPLC-DAD profiles of creamed honey samples with a higher share of additives and a control sample were obtained using Gilson chromatographic set (Gilson Inc., Middleton, WI, USA). The set of apparatus includes a binary gradient pump (Gilson 322), a column thermostat (Knauer, Berlin, Germany), an autosampler with a fraction collector (Liquid Handler GX-271) and a photodiode array detector (DAD, Gilson 172). Poroshell 120, EC C-18, 4.6 × 150 mm column (Agilent Technologies Inc., Santa Clara, CA, USA), maintained at 40 ◦C, was used for separation. A gradient program was applied: 10% B (1.5 min), 10–100% B (1.5–20 min), 100% B (20–25 min), and again 10% B to equilibrate the column (A: 0.1% (*v*/*v*) formic acid in water, B: acetonitrile). A mobile phase flow of 1 mL/min was used and the injection volume of the sample was 20 µL. The chromatograms were recorded at λ = 254, 280, 320, 360 and 520 nm. Polyphenolic compounds were identified by their UV–VIS spectra and by the comparison of their retention time values with analytical standards. Details can be found in Supplementary Materials (Figure S1).

### 3.10. Antioxidant Properties

*DPPH radical scavenging assay*: the total free radical scavenging capacity of honey samples, leaves, and fruit extracts was estimated using the stable DPPH (2,2-diphenyl-1-picrylhydrazyl) radical according to the method previously used in our laboratory for honey analysis [32]. A test solution of honey or fruit extract in the volume of 20 µL was added to 180 µL of methanolic DPPH (0.1 mM), shaken vigorously and kept at room temperature in the dark for 30 min. After incubation, the absorbance of the samples was measured at 517 nm using a microplate reader (Epoch 2, BioTek, Winooski, VT, USA) against methanol as a blank. In the control sample, an appropriate solvent was used instead of the analyzed sample. The capability to scavenge the DPPH radical was calculated using the following Equation (3):

DPPH scavenged (%) = ((A_0_ − A_S_)/A_0_) × 100%, (3)

where A_0_ is absorbance of a blank, and A_S_ is the absorbance of the sample. Results were expressed as Trolox equivalents (TE) using a calibration curve.

*FRAP assay*: the ferric reducing antioxidant power test was determined according to Dżugan et al. (2018) [32]. FRAP reagent was prepared by mixing 300 mM acetate buffer (pH 3.6), 10 mM TPTZ (2,4,6-tripyridyltriazine) in 40 mM HCl and 20 mM FeCl_3_ in the proportion of 10:1:1. The analyzed samples in the volume of 20 µL were added to 180 µL of freshly prepared FRAP reagent in microplate wells. After 10 min of incubation, the absorbance of reaction mixture was measured at 593 nm (Epoch 2) against a blank. For the calibration curve, Trolox was used, and the results were expressed as mmol Trolox equivalents (TE) per 100 g of product.

### 3.11. Antiviral Activity Determination

To determine the antiviral potential of honey the assay described by Miłek et al. (2021) [8] was used. For this purpose, bacteriophage phi6 (DSM 21518) a model organism for enveloped RNA viruses and its host *Pseudomonas syringae* (DSM 21482) were purchased from Leibniz-Institute DSMZ, Deutsche SammLung von Mikroorganismen und Zellkulturen GmbH (Braunschweig, Germany). Bacteriophage phi6 was replicated as follows: 25 mL of TSB (Tryptic Soy Broth, Biomaxima, Poland) was inoculated with a single colony of *P. syringae* and incubated overnight (25 °C, 200 rpm) to the optical density of 0.20–0.25 (OD 600 nm) [68]. Then, 10 µL of phage solution (10^5^ PFU/mL) in SMG buffer (50 mM Tris-HCl pH 7.5, 0.1 M NaCl, 8.1 mM MgSO_4_, 0.01% (*w*/*v*) gelatin) was added to 100 µL of overnight bacterial culture, mixed with 5 mL of a semi-solid medium of TSB (with 0.7% bacteriological agar) and poured onto a Petri dish containing the TSA medium. After overnight incubation at 25 °C, plates were examined for plaque formation and used for phage elution. For this purpose, 5 mL of SMG buffer was added onto the plate and shaken at 100 rpm for 40 min at 25 °C. Then, the mixture was centrifuged at 5000 rpm for 5 min and the supernatant was filtered through 0.22 µm cellulose acetate filters. The filtrate was collected as a virus stock and stored at 4 °C for further analysis.

The antiviral potential of honey with blackberry and raspberry addition was determined by using the double agar overlay plaque method. In Eppendorf tubes, 10 µL of appropriately diluted phage phi6 was mixed thoroughly with 100 µL of 25% water solutions of honey and incubated for 10, 120 min, and 24 h at room temperature (20 ± 2 °C). Additionally, for honey enriched with leaves extracts analyses were determined using 50% concentrations of honey. As a positive control phage in SMG buffer was used. After viral exposure to honey, serial dilutions of samples were prepared in SMG buffer and 10 µL of diluted samples were added to 100 µL of overnight *P. syringae* culture (OD 600 nm = 0.25), then mixed with 5 mL of semi-solid TSB medium (0.7% agar) and poured onto the TSA plate. After 24 h of incubation at 25 °C, plates were examined for plaque formation and the number of bacteriophages per mL of sample was calculated and expressed as log_10_ PFU/mL. The antiviral potential was expressed as a reduction in log_10_ PFU/mL of the analyzed sample in comparison with the control sample (pure bacteriophage).

### 3.12. Antibacterial Properties

The broth microdilution method described by Grabek-Lejko et al. (2018) [69] was used to determine of antibacterial properties of honey samples against Gram-positive bacteria *Staphylococcus aureus* ATCC 25923 and Gram-negative *Escherichia coli* PCM 2561. Overnight bacterial cultures were suspended in phosphate-buffered saline (PBS), pH 7.2, and the turbidity of the suspension (measured at 600 nm, spectrophotometer Hach DR 6000 UV-VIS) was adjusted to around 10^8^ CFU/mL and diluted with a double concentrated Mueller–Hinton broth medium to a final concentration of bacteria around 1 × 10^6^ CFU/mL. Bacterial suspension, in the volume of 100 µL, was mixed with 100 µL of different concentrations of honey samples diluted in water in 100-well microplates and incubated with medium aeration at 37 °C for 24 h in a Bioscreen C apparatus (Oy Growth Curves AB, Ltd., Helsinki, Finland). Positive control was MHB with bacteria, and without honey addition, negative control was pure media MHB. The optical density of the culture was monitored every 1 h at 600 nm. Results were expressed as the percentage of bacterial growth inhibition in comparison with the corresponding positive control after 24 h of incubation.

### 3.13. Assessment of S. aureus Biofilm Formation

The antibiofilm activity of enriched honey was tested by assessing their ability to inhibit biofilm formation and disrupt pre-formed biofilms by *S. aureus* ATCC 25923, according to Coimbra et al. (2022) [70]. For this purpose, *S. aureus* was initially grown in TSB broth overnight at 37 °C. Then, the bacterial suspension was prepared and adjusted to OD600 nm = 1.5 and diluted with TSB medium supplemented with 0.5% of glucose to achieve a final concentration in the wells of around 1 × 10^7^ CFU/mL. Then, 50% (*w*/*v*) honey samples were prepared by dilution in TSB medium supplemented with 0.5% glucose. In total, 100 µL of serially diluted honey samples and 100 µL of bacterial suspension were added to each well of 96-polystyrene plates. For positive control only, the bacterial suspension with medium was used, and for negative control only the culture medium was used. After inoculation, the plates were incubated without shaking at 37 °C for 24 h, and the wells were gently washed twice with 400 µL of water to remove non-adherent cells. Adherent biofilms were fixed with 0.2 mL of methanol for 20 min. After that, wells were air-dried, then stained with 0.1% crystal violet in a volume of 0.2 mL for 10 min. Excess crystal violet was decanted and the wells were washed 4 times with 0.4 mL of water. The stain that was bound to the adherent biomass was resolubilized in 200 µL 0.33% (*v*/*v*) of glacial acetic acid and the absorbance was measured at 595 nm using a microplate reader (Smart Reader 96, Accuris Instruments, Edison, NJ, USA).

To determine the effect of the honeys on preformed biofilms, 24 h biofilms in microtiter plates (prepared as described above) were washed twice with 200 µL of distilled water, then 200 µL aliquots of serially diluted honey samples in TSB were added to each well. The plates were incubated at 37 °C for a further 24 h, then they were stained with crystal violet, and biofilms were assessed as described previously.

### 3.14. Statistical Analysis

All calculations were made in triplicate. For the obtained data, mean values and standard deviations were calculated. The correlations between the obtained parameters were analyzed by Pearson coefficient (r). Significant differences were calculated by two-way analysis of variance followed by NIR Fisher’s test of significant difference (*p* < 0.05). Multidirectional analysis of variance with Wilk’s test was performed to determine the influence of independent factors as well as the interactions between them at the significance level *p* = 0.05. All calculations were made using the Statistica 13.3 software (StatSoft, Tulsa, OK, USA).

## 4. Conclusions

The addition of powdered fruits and leaves of blackberry and raspberry to rape honey strongly increases its functional properties (antioxidant, antiviral and antibacterial potential). Observed improvements were related to honey enrichment with some polyphenols which were slightly higher for blackberry vs. raspberry and for leaves vs. fruits, as well as harvested vs. commercial samples.

Moreover, the higher dose of the *Rubus* additives to honey, the better the antiviral potential for enriched honey was observed against phi 6 bacteriophage—surrogate of the SARS-CoV-2 virus. Better properties of raspberry-enriched honeys could result from the profile of different bioactive components, which was confirmed by the HPTLC and HPLC methods. It seems that non-anthocyanin polyphenols are crucial factors creating the antiviral and antimicrobial activity of enriched honey.

To summarize, the designed novel products based on honey and Rubus spp. leaves or fruit will enrich the assortment of functional food. A honey supplemented with blackberry and raspberry fruits and leaves can be considered an interesting novel functional food, with enhanced antioxidant, antimicrobial and antiviral properties. The new beneficial use of leaves, a by-product of Rubus cultivation, has been demonstrated. The proposed products will allow the leaves to be introduced to the diet in a convenient and organoleptically attractive form. Due to the demonstrated antiviral activity, such products can be used as an adjunct in the treatment of viral diseases. Due to the fact that natural products may differ in chemical profiles even within the same species, the presented results are preliminary and require confirmation in a greater number of raw material variants.

## Figures and Tables

**Table 1:** Total phenolic, flavonoid, and anthocyanin contents, as well as antioxidant capacity of tested plant additives to honey.

		TPC [mg GAE/g d.m.]	TFC [mg QE/g d.m.]	TAC [mg Cy-3-glu/g d.m.]	FRAP [μmol TE/g d.m.]	DPPH [μmol TE/g d.m.]
BH	Leaf	85.37 ± 3.31 ^a^	11.59 ± 0.36 ^a^	-	496.13 ± 11.51 ^a^	295.97 ± 17.02 ^a^
	Fruit	28.68 ± 0.15 ^b^	0.42 ± 0.03 ^b^	4.79 ± 0.01 ^a^	191.69 ± 3.14 ^b^	94.81 ± 0.34 ^b^
BC	Leaf	45.87 ± 0.16 ^c^	4.06 ± 0.08 ^c^	-	283.47 ± 3.84 ^c^	194.19 ± 26.88 ^c^
	Fruit	27.73 ± 0.24 ^b^	0.44 ± 0.02 ^b^	3.79 ± 0.01 ^b^	180.47 ± 1.92 ^b^	86.87 ± 1.36 ^b,d^
RH	Leaf	52.96 ± 0.24 ^d^	13.82 ± 0.12 ^d^	-	276.19 ±1.57 ^b,c^	178.71 ± 0.63 ^c^
	Fruit	20.65 ± 0.00 ^e^	0.25 ± 0.01 ^b^	2.53 ± 0.00 ^c^	125.70 ± 1.22 ^d^	65.33 ± 1.53 ^d^
RC	Leaf	27.57 ± 3.95 ^b^	3.89 ± 0.24 ^c^	-	172.82 ± 1.57 ^b^	109.61 ± 0.63 ^b^
	Fruit	18.97 ± 0.16 ^e^	0.24 ± 0.02 ^b^	2.90 ± 0.00 ^d^	123.36 ± 3.49 ^d^	61.48 ± 1.87 ^d^

‘-’—not determined, ^a,b,c,d,e^—means sharing the same superscript letter in a column do not differ significantly (Tukey’s test, *p* = 0.05).

**Table 2:** Total phenolics and flavonoids contents as well as antioxidant capacity of creamed honeys enriched with *Rubus* sp. leaves and fruits.

		TPC [mg GAE/kg]	TFC [mg QE/kg]	FRAP [mmol TE/kg]	DPPH [mmol TE/kg]
	control	355.65 ± 2.10	91.6 ± 4.32	0.59 ± 0.01	0.53 ± 0.06
BH	+Leaf 0.5%	875.00 ± 46.30	163.36 ± 15.11	3.09 ± 0.25	2.61 ± 0.45
	+Leaf 1%	1550.6 ± 40.87	232.06 ± 0.00	5.85 ± 0.22	6.07 ± 0.08
	+Fruit 1%	650.30 ± 2.10	146.56 ± 8.64	1.88 ± 0.02	2.16 ± 0.35
	+Fruit 4%	1282.73 ± 139.90	245.08 ± 9.43	4.66 ± 0.14	3.94 ± 0.11
BC	+Leaf 0.5%	721.73 ± 23.15	77.86 ± 2.16	2.01 ± 0.09	1.77 ± 0.07
	+Leaf 1%	1400.29 ± 6.31	213.74 ± 4.32	8.05 ± 0.32	4.33 ± 0.11
	+Fruit 1%	940.47 ± 24.40	170.99 ± 4.32	2.64 ± 0.03	2.21 ± 0.55
	+Fruit 4%	1514.88 ± 54.72	319.08 ± 2.16	5.39 ± 0.13	4.22 ± 0.43
RH	+Leaf 0.5%	717.26 ± 4.21	213.74 ± 8.64	2.06 ± 0.09	1.76 ± 0.03
	+Leaf 1%	1449.41 ± 29.46	425.95 ± 6.48	5.17 ± 0.08	5.32 ± 0.11
	+Fruit 1%	552.08 ± 2.10	68.70 ± 2.16	1.55 ± 0.07	1.51 ± 0.37
	+Fruit 4%	1050.59 ± 33.67	204.58 ± 12.95	3.82 ± 0.05	2.62 ± 0.02
RC	+Leaf 0.5%	863.10 ± 29.46	175.57 ± 6.48	2.43 ± 0.17	2.21 ± 0.11
	+Leaf 1%	1385.42 ± 31.57	213.74 ± 8.64	4.71 ± 0.29	3.97 ± 0.30
	+Fruit 1%	601.19 ± 12.63	79.39 ± 3.23	1.55 ± 0.03	1.52 ± 0.52
	+Fruit 4%	1197.92 ± 23.15	285.50 ± 6.48	4.25 ± 0.05	3.21 ± 0.13

All values for enriched honeys differed statistically from the control (*p* < 0.05). Data are presented as mean ± SD (*n* = 6, two sample of each product analyzed in triplicate).

**Table 3:** Correlation matrix for data obtained for creamed honey samples.

	TPC	TFC	FRAP	DPPH
TPC	1			
TFC	0.807	1		
FRAP	0.926	0.682	1	
DPPH	0.951	0.774	0.887	1

## Data Availability

Not applicable.

## Supplementary Materials

The following supporting information can be downloaded at: https://www.mdpi.com/article/10.3390/molecules27154859/s1, Figure S1: HPLC-DAD qualitative profiles of analyzed enriched honey samples; Table S1: The appearance of the obtained honeys enriched with the addition of fruits (left) and leaves (right) of Rubus spp.

## Sample Availability

Samples of the compounds are available from the authors for the limited time.
