# Supplementary material for: Antiviral and Antibacterial Effect of Honey Enriched with Rubus spp. as a Functional Food with Enhanced Antioxidant Properties
Source: Molecules. 2022 Jul 29;27(15):4859. doi: 10.3390/molecules27154859 (PMC9370118; doi:10.3390/molecules27154859)
Supplement: Supplementary file 1 [file molecules-27-04859-s001.zip › molecules-1822559-supplementary.pdf]

Table S1. HPLC-DAD qualitative profiles of analyzed enriched honey samples. BH – blackberry harvested form ecological crop, BC – blackberry commercial, RH – raspberry harvested form ecological crop, RC – raspberry commercial, Control – basic rape honey. The numbers indicate the percentage (w/w) of the additive in creamed honey.

**Peak number**	**Retention time [min]**	**Absorption maxima [nm]**	Tentative **identification**	Control	BH fruit 4%	BH leaf 1%	BC fruit 4%	BC leaf 1%	RH fruit 4%	RH leaf 1%	RC fruit 4%	RC leaf 1%
**1**	2.32	280, 516	cyanidin-3-galactoside*	-	-	-	-	-	-	-	+	-
**2**	3.05	280, 516	cyanidin-3-arabinoside*	-	-	-	+	-	+	-	+	-
**3**	3.50	239, 331, 336sh	unknown	+	+	+	+	+	+	+	+	+
**4**	3.58	279	catechin isomer	-	-	-	+	-	-	+	-	+
**5**	4.67	280, 516	cyanidin-3-glucoside*	-	+	-	-	-	-	-	-	-
**6**	4.85	280	(+)-catechin*	-	+	+	+	+	+	-	+	+
**7**	5.07	280, 516	cyanidin derivative	-	+	-	-	-	-	-	-	-
**8**	5.67	219, 243, 267sh, 328	chlorogenic acid*	-	-	-	-	-	-	+	-	-
**9**	5.95	279	(-)-epicatechin*	-	-	+	+	+	+	-	+	+
**10**	6.25	280	procyanidins	-	+	+	+	+	+	+	+	+
**11**	6.53	280		-	+	+	+	+	+	+	+	+
**12**	6.60	280		-	+	+	+	+	+	+	+	+
**13**	6.83	280		-	+	+	+	+	+	+	+	+
**14**	7.02	253, 367	ellagic acid*	-	-	+	-	+	-	+	-	+
**15**	7.15	266, 348	kaempferol glycoside	+	-	+	-	+	+	+	-	+
**16**	7.28	211, 226, 310	p-coumaric acid*	+	-	+	-	+	-	+	-	+
**17**	7.65	254, 367	rutin*	-	-	+	-	+	-	+	-	+
**18**	7.76	257, 372	isoquercitrin*	-	-	+	-	+	-	+	-	+
**19**	7.90	266, 348	kaempferol glycoside	-	-	+	-	-	-	-	-	+
**20**	7.99	218, 235, 294sh, 323	ferulic acid*	+	-	-	-	-	+	-	-	+
**21**	8.11	266, 348	kaempferol glycoside	-	-	+	-	-	-	+	-	+

**22**	8.44	266, 348	kaempferol-3 glucoside*	-	-	+	-	+	-	+	-	+
**23**	8.71	268, 338	apigenin glycoside	-	-	+	-	-	-	+	-	-
**24**	9.20	230	benzoic acid*	+	+	+	+	+	+	+	+	+
**25**	9.96	266	gallic acid derivative	+	+	+	+	+	+	+	+	+
**26**	10.35	241, 266	unknown	+	+	+	+	+	+	+	+	+
**27**	11.51	268, 338	apigenin*	-	+	+	+	+	-	+	-	-
**28**	11.73	266, 366	kaempferol*	+	-	+	-	+	-	+	-	-
**29**	11.99	292	pinobanksin*	+	+	+	+	+	+	+	+	+
**30**	12.74	278	unknown	+	+	+	+	+	+	+	+	+
**31**	14.43	291	pinocembrin*	+	+	+	+	+	+	+	+	+

* - identification based on comparison with analytical standard, sh – band shoulder

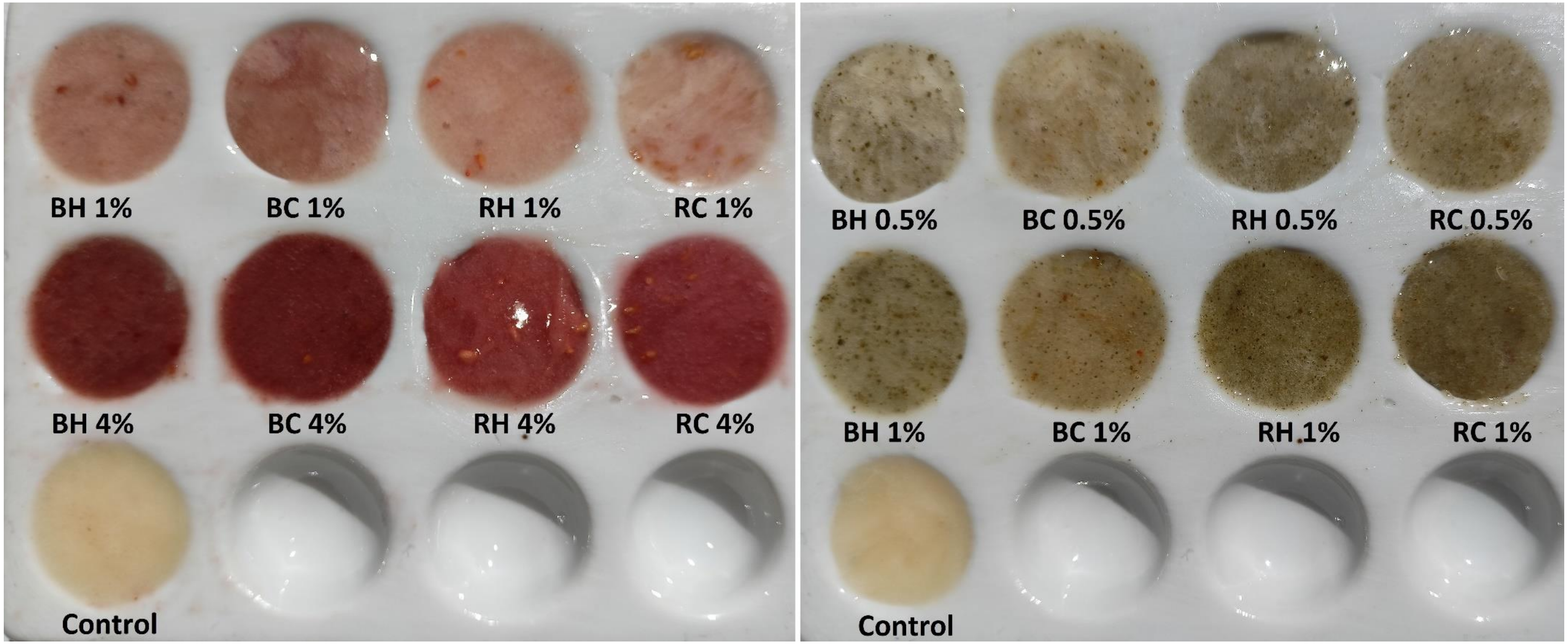

Figure S1. The appearance of the obtained honeys enriched with the addition of fruits (left) and leaves (right) of *Rubus* spp. BH – blackberry harvested form ecological crop, BC – blackberry commercial, RH – raspberry harvested form ecological crop, RC – raspberry commercial, Control – basic rape honey. The numbers indicate the percentage (w/w) of the additive in creamed honey.
